# Clinical Characteristics and Outcome of MPOX Patients Admitted to the Bukavu University Clinics in the Democratic Republic of Congo From July to December 2024: Open Cohort Study

**DOI:** 10.1155/jotm/9981208

**Published:** 2025-06-16

**Authors:** Roland Lwandiko Cibenda, Paul Tshonda Ngongo, Delphin Murhula Katabana, Philippe Bianga Katchunga

**Affiliations:** Department of Internal Medicine, Bukavu University Clinics, Faculty of Medicine, Official University of Bukavu, Bukavu, Congo

**Keywords:** Bukavu, characteristic, MPOX, outcome

## Abstract

**Background:** Several studies have focused on the town of Kamituga, the epicentre of the current MPOX Clade Ib epidemic, in South Kivu Province, in the east of the Democratic Republic of Congo. However, the disease is widespread in several health zones in this province.

**Objectives:** The aim of this study was to describe the clinical characteristics and vital prognosis of admissions for MPOX in the city of Bukavu (21,808 inhabitants/km^2^), in the province of South Kivu.

**Methodology:** Between 1 July and 31 December 2024, MPOX patients were recruited as and when they were admitted to the treatment centre at the Bukavu university clinics. For each patient, clinical characteristics and outcome were sought.

**Results:** Of the 343 patients hospitalized during this study period, 201 (58.6%) were men and 142 (41.4%) were women (*p*=0.001). The median age was 21.0 (11.0–27.7) years. Traders (42.3%) and field workers (24.7%) were more numerous. Contact with an MPOX case (61.2%) was the most frequent mode of contamination. The frequency was 70.2% for elevated white blood cells, 73.0% for elevated c-reactive protein, 37.9% for anemia, 43.1% for genital lesions, 7.0% for pregnancy and 2.6% for HIV. Finally, during the 2400 patient-day observation period, 3 patients subsequently died (0.8%). The incidence of death was 1.25/1000 patient days.

**Conclusion:** The results of this study show that, in the city of Bukavu, MPOX is a disease of young people. Contact with a case of MPOX was the most important mode of contamination, and the professions at risk were trade and field work. Thus, the list of professions at risk of MPOX to be vaccinated must be established according to each region.

## 1. Introduction

Monkeypox virus (MPXV) is a member of the Poxvirus family, the Chordopoxvirus subfamily and the Orthopoxvirus genus [1]. This virus is responsible for a zoonotic disease, monkeypox (MPOX). The disease was first documented in monkeys in 1958 [2] and then in humans in 1972 [3].

Since then, sporadic epidemics have generally occurred in small villages with limited access in forested areas of Central and West Africa, caused by Clade Ia and Clade IIa, respectively [1, 4, 5]. However, since 2003, imported forms of the disease have been reported in several regions of the world [6–9].

In 2017, a mutation in MPXV Clade II, known as Clade IIb, was identified in West Africa [10]. This mutation has significantly increased human-to-human transmission through direct contact and sexual transmission. In April 2024, 95,226 confirmed cases of MPOX were attributed to Clade IIb in 117 countries [11].

In the Democratic Republic of Congo (DRC), in particular, the number of MPOX cases has begun to increase gradually since 2023.

As of 15/12/2024, 9513 (69.0%) of the 13,769 confirmed MPOX cases in Africa had been identified in the DRC [11]. This new epidemic in the DRC is due to MPOXV Clade Ia but also to a new mutation, MPOXV Clade Ib [12, 13]. The epicentre of this new mutation is the province of South Kivu, in the east of the DRC [12, 13]. MPOXV Clade Ib has also been detected in countries bordering the province of South Kivu (Burundi, Rwanda, and Uganda) [11]. From January to May 2025, cases of MPOXV Clade Ib increased to 31,899. Imported cases of the disease have been reported in the United Kingdom, Germany, France, Switzerland, Thailand, and the United Arab Emirates [14].

Several authors have already reported on the clinical and epidemiological profile of MPOX documented in the mining town of Kamituga (260,000 inhabitants) in South Kivu province, the starting point of the MPOXV Clade Ib epidemic [15–17]. Sexual transmission was the main mode of contamination.

However, cases of MPOX are currently being reported in several health zones in South Kivu province, particularly in the city of Bukavu, the province's economic and administrative capital. The city of Bukavu currently has a population of 1,308,470 over an area of 60 km^2^, giving a density of 21,808 inhabitants per km^2^. The socio-economic profile of the city of Bukavu could certainly have an impact on the epidemiological profile of MPOX.

The aim of this study is to describe the clinical characteristics and prognosis of MPOX patients admitted to the MPOX treatment centre at the Bukavu University Clinics (CUB) from 1 July 2024 to 31 December 2024.

## 2. Methods

This study was an open cohort. It took place in the MPOX treatment centre of the CUB, a university hospital of the Official University of Bukavu located in the city of Bukavu. The CUB is at the crossroads of 3 communes in the city of Bukavu: Ibanda, Kadutu and Bagira.

The study period ran from 1 July 2024 to 31 December 2024.

The study population consisted of MPOX patients confirmed by molecular biology. These patients were recruited as they were admitted to the CUB MPOX treatment centre, regardless of age and sex. Each patient was followed in hospital until discharge, regardless of outcome.

Data were collected anonymously and confidentially. The privacy and personality of the participants were protected in accordance with the Declaration of Helsinki. The protocol for this study was accepted by the ethics committee of the official University of Bukavu (UOB/CEM/072/2024).

### 2.1. Data Collection

A team of doctors and nurses were trained in the management of MPOX patients and in data collection according to the guidelines of the DRC's National Ministry of Health [18].

On admission to the CUB treatment centre, the following data were collected for each patient: demographic parameters (age, sex, place of residence), socio-economic level (profession and level of education), clinical parameters (symptoms on admission, mode of contamination and type and extent of lesions). Based on these clinical parameters, a severity score (mild, moderate, severe or critical) was established. Co-morbidities and other predispositions were also investigated (HIV infection, diabetes mellitus, hypertension, cancer, syphilis, malaria, malnutrition and pregnancy).

Following clinical examination, suspected cases according to the case definition were selected [18].

MPOX patients were classified according to the number of lesions as mild (< 25 skin lesions) or moderate (25–99 skin lesions), severe (100–250 skin lesions), or critical (> 250 skin lesions) [16].

The medical treatment of each patient followed the recommendations of the DRC Ministry of Health [18].

### 2.2. Biological Analyses

In suspected cases of MPOX, a superficial skin lesion was swabbed and rubbed. The GeneXpert system was the diagnostic test used in the provincial laboratory.

Nevertheless, the first suspected cases of MPOX in South Kivu province were confirmed by RT-PCR. Genetic analysis has shown a new mutation in MPXV clade I, called Clade Ib [12, 13].

A venipuncture was also performed for hematological, biochemical and serological tests (blood count, c-reactive protein (CRP), creatinine, glycaemia, HIV and *Treponema pallidum* serology).

### 2.3. Statistical Analysis

The distribution of the variables was tested for normality using the Kolmogorov–Smirnov test. Thus, the data are presented by the median (interquartile range) or the relative frequency in percent. The non-parametric Kruskal–Wallis test was used to compare several medians.

The Chi-square test was used to compare categorical variables. But when the numbers were low, less than 30, we used Fisher's exact test.

The rate of progression to death was calculated for the entire group by dividing the number of patients who died by the number of patient-day of observation. The case fatality rate was obtained by dividing the number of deaths by the total number of patients.

A *p* value < 0.05 was considered to be significant.

MedCalc version 18.11 software was used for statistical analyses.

## 3. Results

### 3.1. Demographic Parameters

**Figure 1:**
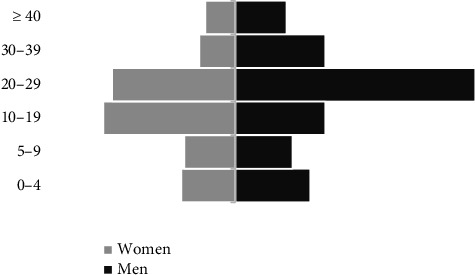
Age pyramid of MPOX patients.

**Figure 2:**
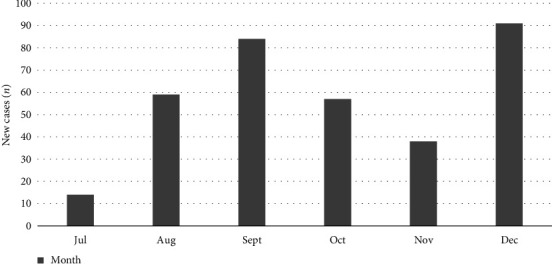
Number of admissions for MPOX between July and December 2024.

Table 1 and Figures 1 and 2, respectively, show the general characteristics, the age pyramid of the patients studied and the number of new cases between July and December 2024. Between 1 July 2024 and 31 December 2024, three hundred and forty-three (343) MPOX patients were hospitalised in the CUB treatment centre, including 201 (58.6%) men and 142 (41.4%) women (*p*=0.001).

The median age was 21.0 (11.0–27.7) years. The 20–29 age group was significantly more represented (35.3%), dominated by men (39.8% vs. 28.9%; *p*=0.03), followed by the 10–19 age group (21.6%), dominated by women (31.0% vs. 14.9%; *p*=0.0004). Only 27 (7.9%) patients were aged 40 or over.

### 3.2. Socio-Economic Parameters

The socio-economic parameters of the patients studied are shown in Table 1. In the whole group, 235 (68.5%) had a high level of education. Among the workers, tradesmen (42.3%) and field workers (24.7%) were the most represented. Only 8 (8.2%) patients were medical staff.

244 (71.2%) MPOX patients came from the two most densely populated communes (Ibanda and Kadutu).

### 3.3. Transmission Mode

Table 2 shows the mode of transmission of the disease. 210 (61.2%) patients reported having been in contact with a case of MPOX, 108 (31.5%) patients had recent sexual contact and 66 (19.2%) patients reported both types of contact. Recent sexual contact was more frequent in men than in women (36.8% vs. 23.9%; *p*=0.01).

### 3.4. Clinical Characteristics of MPOX Patients

Table 3 shows the Clinical parameters on admission and the characteristics of skin lesions of hospitalized MPOX patients. All patients (100.0%) had skin lesions, 315 (91.8%) had pruritus, 258 (75.2%) had fever, 102 (29.7%) had dysphagia and 81 (23.6%) had lymphadenopathies.

**Figure 3:**
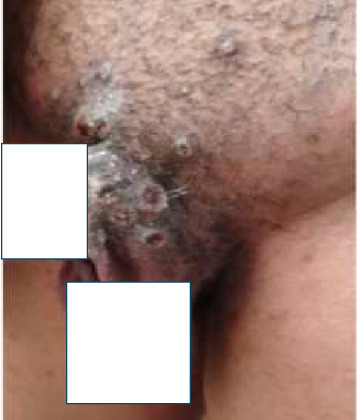
Skin lesions on the genitals of a patient.

Genital skin lesions were present in 148 (43.1%) patients (Figure 3). The genitals were the site of the first skin lesion in 79 (23.0%) patients.

**Figure 4:**
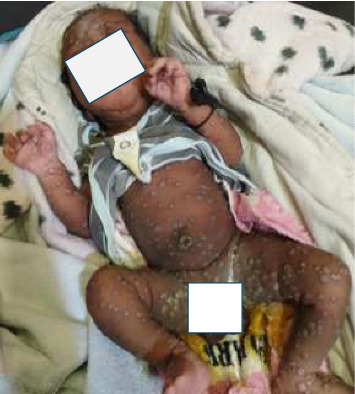
Critical skin lesions in a newborn baby.

Severe/critical skin lesions were present in 72 (21.0%) patients (Figure 4).

There were no significant differences between the sexes in the distribution of injuries.

### 3.5. Biological Parameters

Some of the biological parameters analyzed in 269 patients are shown in Table 1. On admission, the median was 11,724 (8765–13,055)/mm^3^ for white blood cells, 21.0 (7.0–32.0) mg/L for c-RP and 12.0 (9.0–13.0) mg/dL for haemoglobin. 70.2% of patients had WBC > 10,000/mm^3^, 73.0% had CRP > 10 mg/L and 37.9% had anaemia. Anaemia was significantly more common in men than in women (46.2% vs. 26.1; *p*=0.0009).

### 3.6. Comorbidities and Complications

Table 3 shows the co-morbidities and complications present in the patients. 54 (15.7%) MPOX patients had Syphilis, 9 (2.6%) MPOX patients were HIV positive and 10 (7.0%) female patients were pregnant.

The most frequent complications were dermatitis (69.4%), anaemia (37.9%) and skin abscesses (13.7%) a bronchopulmonary infection. Two infants (0.6%) developed encephalitis.

### 3.7. Vital Prognosis

In the present study, over the 2400 patient-day observation period, the incidence of death among the 343 MPOX patients at baseline was 1.25/1000 patient-day. The fatality rate was 0.8%.

The patients who died were two infants aged < 1 year and a 49-year-old adult (Table 4). They were all male and transferred from peripheral centres. Their skin lesions were severe/critical and all had 3 complications.

## 4. Discussions

This study is the first in South Kivu province to describe the clinical features of MPOX in the city of Bukavu, an urban environment. Available studies in this region are rare and have focused on the town of Kamituga, the epicentre of the current MPOX epidemic [15–17].

This study led to the following major observations:

The first observation shows that the median age of MPOX patients hospitalised at the CUB was 21 years, and the predominant age group was between 20 and 29 years. Our results corroborate those from the town of Kamituga, where the median age of MPOX patients was 22 (18–29) years [15]. The predominance of the disease in subjects aged less than 40 years is explained by a progressive loss of community immunity secondary to the cessation of vaccination against human smallpox around 1980 [19]. However, MPOX patients in Western countries (median age: 38 years) [9]. This difference could be explained by a younger age pyramid in Sub Saharan Africa compared with the Western region.

The second observation shows that contact with an MPOX case was the most important mode of contamination (61.2% of cases) in the city of Bukavu, while recent sexual contact was found in 31.5% of cases. These results are quite different from those obtained in the town of Kamituga, where 88.4% of cases were probably due to sexual contact with sex workers [17]. Admittedly, sexual contact is the basis of intimate contact between two individuals, hence the high risk of contamination. However, in our study, the genitals were the site of the first skin lesion in 23.0% of patients, suggesting that MPOX is a sexually transmitted disease. This observation is consistent with that found by other authors in other parts of the world. Thornhill et al. found that sexual contact was the mode of transmission in 95.0% of MPOX patients in Europe [9].

The clinical manifestations were identical in the different studies. However, genital lesions were more frequent in studies in which sexual transmission was the main mode of contamination, notably in Kamituga (79.0%) [14] and Europe (73.0%) [9], compared with this study in which they were identified in 43.1% of cases.

Encephalitis, a serious but rare complication of MPOX, was observed in two infants who subsequently died. The neurological complications of MPOX are common and have been described by several authors [20].

It should also be emphasised that 70% of patients had an inflammatory syndrome marked by leukocytosis and elevated c-RP. This inflammatory state in more than three-quarters of hospitalised patients underlies the severity of the cases, particularly sepsis or bacterial infection of the skin lesions. Curiously, all three deceased cases had systemic inflammatory response syndrome (fever, leukocytosis, elevated CRP) associated with multiorgan failure (sepsis) and a critical severity score. This observation is consistent with the literature [21]. Anaemia was very common among hospitalised MPOX patients in this study (37.9%), particularly among men. These results corroborate those of other authors. Indeed, Mbelambela et al. noted that 40.6% of MPOX patients had anaemia in the Katako-Kombe Health Zone in the DRC [22]. The high frequency of anaemia in men has also been described by other authors [23]. The reason for this observation is not known.

In this study, the professions at risk were mainly trade and field work, in contrast to Kamituga and Europe, where sex workers were most at risk [9, 15]. This observation should lead us to reconsider the list of professions at risk according to each region. Ultimately, vaccination should be directed at all professions where human contact is very important, not just sex workers.

Finally, in this study, the case fatality rate was 0.8% and the incidence of death was estimated at 1.25/1000 patient days, compared with 32/1000 patient days for COVID-19 in the same city of Bukavu [24]. It should be noted that all three MPOX patients who died were transferred from peripheral centres, including two infants less than a year old. This suggests that the case fatality rate for MPOX patients residing in the city of Bukavu was zero. Our results are similar to those of other authors. The case fatality rate in the town of Kamituga was 1.0% (4 deaths out of 371 MPOX patients) [17]. In Europe, no deaths were reported among 528 MPOX patients [9]. At national level in the DRC, the case fatality rate varies from 0.0% to 5.6% (Ministry of Health/DRC). Our results confirm that the case-fatality rate of MPOX is low and would certainly be influenced by the quality of management and the young age of the patients.

The results of this study must be interpreted in the light of its limitations. Firstly, the data for this study were collected using the DRC Ministry of Health's data collection form. This form is very limited, which explains the lack of certain data, particularly biological data. In addition, there was the possibility of memory bias secondary to the participants' responses.

Finally, the longitudinal approach would have made it possible to establish a causal link between the death and the supposed factors indicating a poor prognosis. However, the small sample size reduces the statistical power in this study and makes it impossible to analyze the predictors of death in this study.

In this study, post-infectious complications, particularly neurological complications, could not be investigated due to the short duration of the study. Longitudinal follow-up would have made it possible to establish a causal link between certain complications such as Guillain–Barré syndrome, which is relatively common in the region, and MPOX.

## 5. Conclusion

This study is the first to have been carried out in the city of Bukavu. The results of this study show that MPOX is a disease of young people, with contact with a case of MPOX being the most important mode of contamination, and that the professions at risk were trade and field work. The list of occupations at risk should therefore be considered on a regional basis.

## Figures and Tables

**Table 1:** General characteristics of MPOX patients studied.

	Whole group	Men	Women	*p*
Number, *n* (%)	343 (100.0)	201 (58.6)	142 (41.4)	0.001
Age (years), med (EIQ)	21.0 (11.0–27.7)	22.0 (11.0–29.0)	18.0 (10.0–25.0)	0.006
< 5 years, *n* (%)	43 (12.5)	25 (12.4)	18 (12.7)	0.970
5–9 years, *n* (%)	36 (10.5)	19 (9.5)	17 (12.0)	0.450
10–19 years, *n* (%)	74 (21.6)	30 (14.9)	44 (31.0)	0.0004
20–29 years, *n* (%)	121 (35.3)	80 (39.8)	41 (28.9)	0.030
30–39 years, *n* (%)	42 (12.2)	30 (14.9)	12 (8.5)	0.070
≥ 40 years, *n* (%)	27 (7.9)	17 (8.5)	10 (7.0)	0.610
WBCs (/mm^3^), med (EIQ), (*n* = 269)	11,724 (8765–13,055)	11,535 (6521–13,030)	11,886 (11,050–13,200)	0.096
c-RP (mg/L), med (EIQ), (*n* = 269)	21.0 (7.0–32.0)	19.5 (4.0–32.0)	23.0 (12.0–32.5)	0.443
Hb, med (EIQ), (*n* = 269)	12.0 (9.0–13.0)	12.0 (10.0–13.0)	12.0 (9.0–13.0)	0.119
WBCs > 10,000/mm^3^, (%)	189 (70.2)	101 (63.9)	88 (79.8)	0.008
Hb < 13 (H)/< 12 (F) gr/dL, (%)	102 (37.9)	73 (46.2)	29 (26.1)	0.0009
c-RP > 10 mg/L, (%)	196 (73.0)	108 (68.9)	88 (79.2)	0.070

*Level of study, n (%)*				
Illiterate	54 (15.7)	32 (15.9)	22 (15.5)	0.920
Primary	54 (15.7)	25 (12.4)	29 (20.4)	0.040
Secondary	157 (45.8)	89 (44.3)	68 (47.9)	0.510
University	78 (22.7)	55 (27.4)	23 (16.2)	0.010

*Occupation (n = 97), n (%)*				
Trader	41 (42.3)	25 (35.2)	16 (61.5)	< 0.0001
Field worker	24 (24.7)	21 (29.6)	3 (11.5)	0.0001
Bureaucrat	13 (13.4)	10 (14.1)	3 (11.5)	0.480
Teacher	11 (11.3)	8 (11.3)	3 (11.5)	0.950
Medical staff	8 (8.2)	7 (9.9)	1 (3.8)	0.030

*Municipality of residence, n (%)*				
Ibanda	108 (31.5)	62 (31.0)	46 (32.4)	0.780
Kadutu	136 (39.7)	78 (39.0)	58 (40.8)	0.730
Bagira	60 (17.5)	35 (17.5)	25 (17.6)	0.980
Transferred from a peripheral centre	39 (11.4)	26 (12.9)	13 (9.2)	0.280

Abbreviations: c-RP = c-reactive protein, Hb = haemoglobin, WBCs = white blood cells.

**Table 2:** Transmission mode.

	Whole group	Men	Women	*p*
Contact with a case of MPOX	210 (61.2)	127 (63.2)	83 (58.5)	0.370
Recent sexual contact	108 (31.5)	74 (36.8)	34 (23.9)	0.010
Contact with 1 MPOX case and recent sexual contact	66 (19.2)	48 (23.9)	18 (12.7)	0.009
Not defined	91 (26.5)	48 (23.9)	43 (30.3)	0.180

**Table 3:** Clinical parameters on admission.

Clinical parameters	Whole group *n* (%)	Men *n* (%)	Women *n* (%)	*p*
Skin lesions	343 (100.0)	201 (100.0)	142 (100.0)	—
Pruritus	315 (91.8)	187 (93.0)	128 (90.1)	0.330
Fever	258 (75.2)	155 (77.1)	103 (72.5)	0.330
Dysphagia	102 (29.7)	67 (33.3)	35 (24.6)	0.080
Lymphadenopathies	81 (23.6)	55 (27.4)	26 (18.3)	0.050

*Site of the first lesion*				
Hands (fingers or wrists)	88 (25.7)	50 (24.9)	38 (26.8)	0.690
Trunk	86 (25.1)	46 (22.9)	40 (28.2)	0.260
Face	79 (23.0)	47 (23.4)	32 (22.5)	0.840
Genitals	79 (23.0)	52 (25.9)	27 (19.0)	0.130
Mouth lips	11 (3.2)	6 (3.0)	5 (3.5)	0.790^∗^

*Final location of lesions*				
Genitals	148 (43.1)	93 (46.3)	55 (38.7)	0.160
Other location	195 (56.9)	108 (53.7)	87 (61.3)	0.160

*Severity of skin lesions*				
Middle	107 (31.2)	51 (25.4)	56 (39.4)	0.005
Moderate	164 (47.8)	106 (52.7)	58 (40.8)	0.030
Severe/critical	72 (21.0)	44 (21.9)	28 (19.7)	0.620

*Comorbidities*				
Syphilis	54 (15.7)	33 (16.4)	21 (14.8)	0.680
Malaria	24 (7.0)	12 (6.0)	12 (8.5)	0.390^∗^
Pregnancy	10 (2.9)	—	10 (7.0)	—
HIV	9 (2.6)	5 (2.5)	4 (2.8)	1.000^∗^
Arterial hypertension	6 (1.7)	3 (1.5)	3 (2.1)	0.690^∗^
Diabetes mellitus	2 (0.6)	0 (0.0)	2 (1.4)	0.170^∗^
Cancer	0 (0.0)	0 (0.0)	0 (0.0)	—

*Complications*				
None	72 (21.0)	34 (16.9)	38 (26.8)	0.020
Dermatitis	238 (69.4)	146 (72.6)	92 (64.8)	0.120
Anaemia (*n* = 269)	102 (37.9)	73 (46.2)	29 (26.1)	0.0009
Skin abscesses	47 (13.7)	36 (17.9)	11 (7.7)	0.007
Broncho-pneumonia	20 (5.8)	18 (9.0)	2 (1.4)	0.003^∗^
Digestive disorders	4 (1.2)	2 (1.0)	2 (1.4)	1.000^∗^
Acute malnutrition	4 (1.2)	2 (1.0)	2 (1.4)	1.000^∗^
Eye damage	2 (0.6)	1 (0.5)	1 (0.7)	1.000^∗^
Encephalitis	2 (0.6)	2 (1.0)	0 (0.0)	0.510^∗^

^∗^The Fisher exact test was used.

**Table 4:** Clinical characteristics of MPOX patients who subsequently died.

	1^st^ case	2^nd^ case	3^rd^ case
Age (years)	< 1	1	49
Sex	M	M	M
Place of residence	Out of town	Out of town	Out of town
Contact with a MPOX case	No	No	Yes
Recent sexual contact	No	No	Yes
Genital lesions	No	No	Yes
Severity score	Critical	Severe	Critical
Complications	Dermatitis/pneumonia/encephalitis	Dermatitis/pneumonia/encephalitis	Skin abscesses/dermatitis/pneumonia/
Immune deficiency	No	No	Taking corticosteroids
WBCs (mg/dL)	12,332	15,200	11,203
c-RP (mg/L)	29	30	21
Hb (gr/dL)	12	12	9
Duration of hospitalisation (days)	6	8	17

Abbreviations: c-RP = c-reactive protein, Hb = haemoglobin, WBCs = white blood cells.

## Data Availability

The data that support the findings of this study are available on request from the corresponding author. The data are not publicly available due to privacy or ethical restrictions.
